# Gastric Small-Cell Carcinoma Found on Esophagogastroduodenoscopy: A Case Report and Literature Review

**DOI:** 10.1155/2013/475961

**Published:** 2013-04-04

**Authors:** Natassja Frances, Simon B. Zeichner, Michael Francavilla, Mike Cusnir

**Affiliations:** ^1^Nova Southeastern College of Osteopathic Medicine, 3301 College Avenue, Fort Lauderdale, FL 33314, USA; ^2^Department of Internal Medicine, Mount Sinai Medical Center, 4300 Alton Road, Miami Beach, FL 33137, USA; ^3^Department of Radiology, Mount Sinai Medical Center, FL, USA; ^4^Department of Hematology/Oncology, Mount Sinai Medical Center, FL, USA

## Abstract

*Introduction*. Characterized as an undifferentiated, neuroendocrine tumor arising from totipotent stem cells, small-cell carcinoma (SCC) most commonly arises from the lung. Extrapulmonary small-cell carcinomas (ESCC) are rare and account for only four percent of SCC. Gastric ESCC, more commonly seen in Japanese male patients in their seventh decade of life, accounts for approximately 0.1 percent of ESCC. *Case Presentation*. A 75-year-old Hispanic male presented with a several week history of worsening epigastric pain with nausea and vomiting. Computer tomography (CT) of the abdomen and pelvis showed a large heterogeneous mass involving the posterior gastric wall with diffuse extension into the gastric cardia. Esophagogastroduodenoscopy (EGD) revealed a large fungating mass in the lesser curvature of the stomach. Biopsy of the mass revealed small-cell carcinoma of the stomach. The patient was diagnosed with extensive/stage 4 disease and started on chemoradiation. *Discussion*. Our case, of a very rare condition highlights, the importance of recognizing atypical pathologic diagnoses. More research will need to be conducted with GSCC patients in order to better characterize disease pathogenesis, genetic mutations, and optimal disease management. The hope is to identify biomarkers that will identify patients earlier in their disease course when cure is possible.

## 1. Introduction

Characterized as an undifferentiated, neuroendocrine tumor arising from totipotent stem cells, small-cell carcinoma (SCC) most commonly arises from the lung. Extra pulmonary small-cell carcinomas (ESCC), described in the urinary bladder, prostate, esophagus, stomach, colon and rectum, gallbladder, larynx, salivary glands, cervix, and skin, are rare and account for only four percent of SCC [1]. Gastric ESCC, more commonly seen in Japanese male patients in their seventh decade of life, accounts for approximately 0.1 percent of ESCC [2, 3].

Gastric ESCCs are large tumors, with histologic features similar to those of small-cell lung carcinoma (SCLC) [4], and typically arise in the upper one-third of the stomach [4, 5]. Gastric ESCCs have an aggressive natural history that is characterized by early, widespread metastases. Due to its rarity and the inability to perform randomized clinical trials, the optimal course of therapy is not well defined. The initial management of patients is patterned after SCLC and other tumor types arising from the stomach. Even with early diagnosis, the overall prognosis is poor, with less than 15 percent of patients surviving five years [6].

We report a case of a 75-year-old Hispanic male diagnosed with gastric small-cell carcinoma.

## 2. Case Presentation

**Figure 1:**
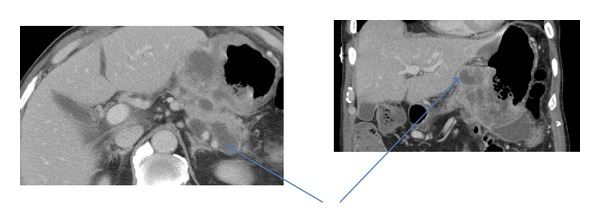
Computer tomography (CT) of the abdomen and pelvis showed a large heterogeneous mass involving the posterior gastric wall with diffuse extension into the gastric cardia. Two irregular hypodensities measuring 1.1 cm and 1.3 cm were identified in the inferior left hepatic lobe.

**Figure 2:**
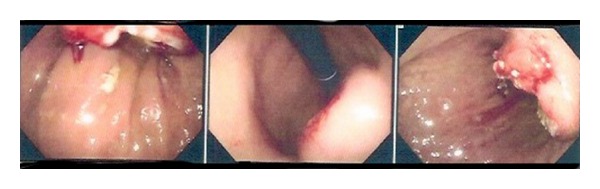
Esophagogastroduodenoscopy (EGD) revealed a large fungating mass in the lesser curvature of the stomach.

**Figure 3:**
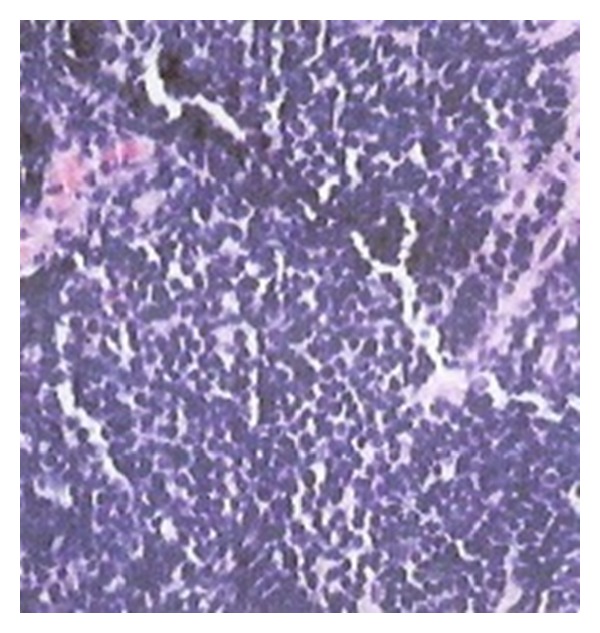
Biopsy of the mass revealed small-cell carcinoma of the stomach.

**Figure 4:**
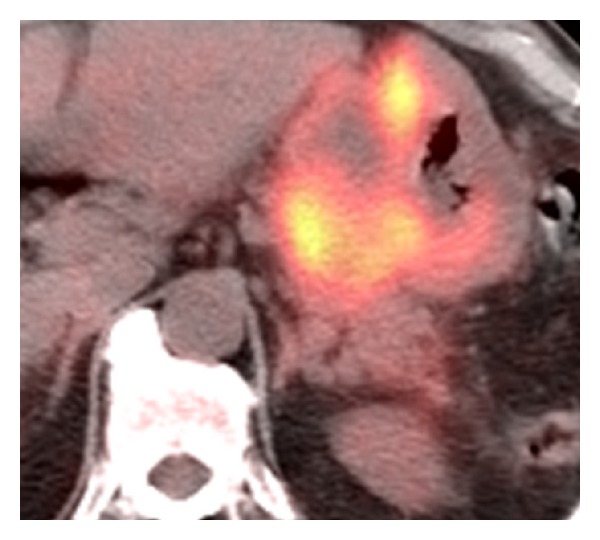
Staging positron emission tomography/computer tomography (PET/CT) of the chest, abdomen, and pelvis revealed fludeoxyglucose (FDG) uptake along the gastric lumen with maximum standardized uptake value (SUV) of 6.5 and increased uptake along the mesentery and cecum with maximum SUV of 5.6 and 5.8, respectively.

A 75-year-old Hispanic male presented with a several week history of worsening epigastric pain with nausea and vomiting. His past medical history was significant for type II diabetes mellitus, hypertension, hypercholesterolemia, and osteoarthritis. He had a 40-pack-year smoking history but had quit thirty years ago. He was afebrile with stable vital signs. Physical exam was notable for tenderness upon palpation in the epigastric region without abdominal distention. Pertinent laboratory studies included white blood cell count 10.2 × 10^3^/*μ*L, hemoglobin 10.8 g/dL, hematocrit 32.7%, mean corpuscular volume 90.5 fL, platelet count 234 × 10^3^/*μ*L, lactate dehydrogenase 143 U/L, chromogranin A, ECL < 1.0 ng/mL (normal range 1.9–15.0), gastrin 255 pg/mL (normal range < 100), carcinoembryonic antigen < 0.5 ng/mL, and cancer antigen 125 (CA-125 < 21 U/mL). Computer tomography (CT) of the abdomen and pelvis showed a large heterogeneous mass involving the posterior gastric wall with diffuse extension into the gastric cardia. Two irregular hypodensities measuring 1.1 cm and 1.3 cm were identified in the inferior left hepatic lobe (Figure 1). Esophagogastroduodenoscopy (EGD) revealed a large fungating mass in the lesser curvature of the stomach (Figure 2). Biopsy of the mass revealed small-cell carcinoma of the stomach (Figure 3) with chronic active *Helicobacter pylori* gastritis. The tumor cells were positive for cytokeratin AE1/AE3 by immunohistochemistry (IHC) and negative for CD3, CD20, CD45, and S100. Staging positron emission tomography/computer tomography (PET/CT) of the chest, abdomen, and pelvis revealed fludeoxyglucose (FDG) uptake along the gastric lumen with maximum standardized uptake value (SUV) of 6.5 and increased uptake along the mesentery and cecum with maximum SUV of 5.6 and 5.8, respectively (Figure 4). 

The patient was diagnosed with extensive/stage 4 disease and started on combination chemotherapy. The patient received four cycles of cisplatin 80 mg/m^2^ on day 1 every 3 weeks and etoposide 100 mg/m^2^/day I.V. on days 1, 2, and 3 every 3 weeks for 4 cycles [7]. He also received 4500 cGy in 25 fractions with the use of intensity-modulated radiation therapy (IMRT). The patient had a partial response to chemoradiation therapy, but his treatment course was complicated by multiple admissions to the hospital for profound pancytopenia, pneumonia, upper gastrointestinal bleeding, and new onset atrial fibrillation. 

**Figure 5:**
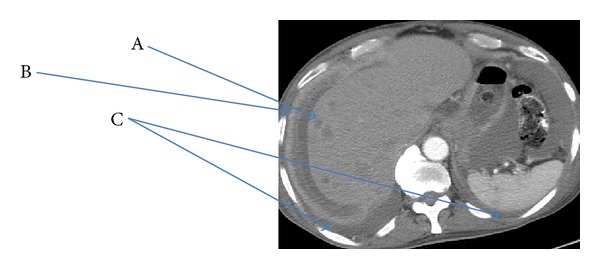
Seven months after initial presentation, a repeat CT of the abdomen showed progression of extensive metastatic disease, numerous hepatic metastases, ascites, and bilateral pleural effusions.

Seven months after initial presentation, the patient was admitted again to the hospital for intractable abdominal pain and shortness of breath. A repeat computed tomography of the abdomen showed progression of extensive metastatic disease, numerous hepatic metastases, ascites, and bilateral pleural effusions (Figure 5). As per the patient's and family's request, palliative measures were instituted and the patient died several weeks later.

## 3. Discussion

Our case of a very rare condition brings to light many interesting issues related to the presentation, diagnosis, staging, and treatment of gastric small-cell carcinoma. 

### 3.1. Epidemiology/Presentation

The risk factors for GSCC are similar to those of non-SCC subtypes (nitrates, *Helicobacter pylori* infection, etc.) and similar to gastric adenocarcinoma, there is a higher incidence among Japanese patients [2]. The initial clinical manifestations of GSCCs are often due to loco-regional disease and thus are indistinguishable from other histologic types arising from the same site. Symptoms can be due to the presence of a mass, ulceration, and bleeding, invasion of adjacent structures, or systemic manifestations of malignancy [1, 5, 8]. GSCC can present with paraneoplastic syndromes, as occurs with small-cell lung cancer (SCLC), and secrete ectopic hormones, such as parathyroid hormone, antidiuretic hormone, calcitonin, or serotonin. Therefore, patients may present with various laboratory abnormalities related to hormone release including hyponatremia, hypokalemia, or hypercalcemia. GSCC most often metastasizes to the liver, and patients may present with signs or symptoms related to disease metastasis. 

### 3.2. Diagnosis

**Figure 6:**
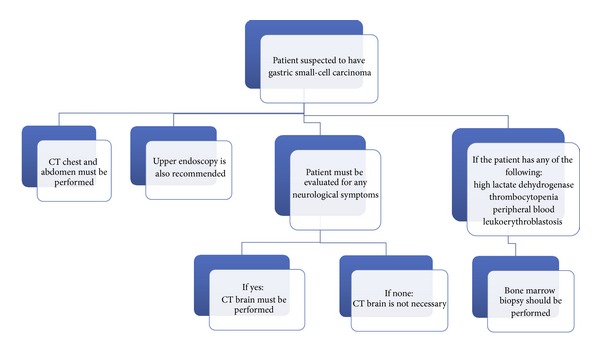
Flowchart to describe staging workup for gastric small-cell carcinoma.

Upper gastrointestinal endoscopy is the preferred procedure for initial evaluation (Figure 6), but barium contrast studies may be useful in detecting the presence of a mass. Although the diagnosis is often made by punch biopsy, the differentiation from lymphoma, poorly or undifferentiated carcinoma, and other tumor types is difficult. GSCC has the same light microscopic appearance as SCLC, with sheets and nests of round fusiform cells with little cytoplasm, granular nuclear chromatin, and neurosecretory granules. The tumors have significant amount of necrosis and a high proliferative rate. IHC staining with neuron-specific enolase, chromogranin, grimelius, and TTF-1 is often helpful in making the diagnosis [1, 8]. 

GSCCs are often found in conjunction with other tumor types, and the presence of a small-cell component usually determines the biologic aggressiveness. The two types of gastric small-cell carcinomas described are pure-type and composite-type. The pure-type contains only histologic features of small-cell carcinoma, whereas the composite-type contains a combination of small-cell carcinoma with one other histologic type, such as adenocarcinoma or squamous cell carcinoma [3]. Han et al., using mutational analysis and immunohistochemistry, showed that the composite-type may be of monoclonal origin [9].

### 3.3. Staging

Upon diagnosis, patients should be evaluated to rule out a primary SCLC or evidence of distant metastases with the use of PET-CT. Although there is no specific staging system for gastric small-cell carcinoma, the tumor-node-metastasis (TNM) system [10] and the Veterans' Administration Lung Study group (VALSG) system are two systems currently being used to stage patients. In the TNM system, patients are approached similar to gastric tumors of different histology and are given a stage from one to four based on tumor and nodal involvement. However, in the VALSG system, patients are approached similar to SCLC and are divided based on limited or extensive disease. Limited disease is defined as a localized tumor within one region with or without regional lymphadenopathy, whereas extensive disease is defined as a tumor that is outside the locoregional boundaries. 

### 3.4. Treatment

Although most patients present with advanced disease, total gastrectomy and lymph node dissection with radiation therapy may provide locoregional control and subsequent long-term survival in isolated cases [11–14]. Local and distant recurrence is common secondary to micrometastasis, and adjuvant systemic chemotherapy is generally recommended for all patients. No randomized clinical trials have been conducted comparing agents for GSCCs and the management of systemic disease with chemotherapy is patterned after SCLC, with the use of platinum-based regimens in combination with etoposide. Although objective responses are commonly observed, most of them are partial and of short duration [15, 16]. In Japan, regimens used for gastric adenocarcinoma, like tegafur and uracil or fluorouracil and mitomycin, have generated some success [14]. For patients with liver involvement, radiofrequency interstitial thermal ablation and/or intraarterial hepatic chemotherapy have been proposed as possible alternative therapeutic options [12]. Prophylactic cranial irradiation is not routinely recommended because of a lower rate of brain metastases in GSCC, as compared to SCLC [17, 18]. Despite the use of multimodality treatment, the overall prognosis of GSCC is poor [1, 14]. In one series, 34 of 67 patients died within one year, and only five patients (7.5 percent) survived two years [14]. 

## 4. Conclusion

Our case of a very rare condition highlights the importance of recognizing atypical pathologic diagnoses. More research needs to be conducted with GSCC patients in order to better characterize disease pathogenesis, genetic mutations, and an optimal disease management. The hope is to identify biomarkers that will diagnose patients earlier in their disease course when cure is possible.
